# Metabolomic profiling of CSF in multiple sclerosis and neuromyelitis optica spectrum disorder by nuclear magnetic resonance

**DOI:** 10.1371/journal.pone.0181758

**Published:** 2017-07-26

**Authors:** Hyun-Hwi Kim, In Hye Jeong, Ja-Shil Hyun, Byung Soo Kong, Ho Jin Kim, Sung Jean Park

**Affiliations:** 1 College of Pharmacy and Gachon Institute of Pharmaceutical Sciences, Gachon University, Incheon, Korea; 2 Department of Neurology, Research Institute and Hospital of National Cancer Center, Goyang, Korea; National Research Council of Italy, ITALY

## Abstract

Multiple sclerosis (MS) and neuromyelitis optica spectrum disorder (NMOSD) are inflammatory diseases of the central nervous system. Although several studies have characterized the metabolome in the cerebrospinal fluid (CSF) from MS and NMOSD patients, comparative analyses between them and between the relapse and the remission of each disease have not been performed. Both univariate and multivariate analyses were used to compare ^1^H-NMR spectra of CSF from MS, NMOSD, and healthy controls (HCs). The statistical analysis showed alterations of eight metabolites that were dependent on the disease. Levels of 2-hydroxybutyrate, acetone, formate, and pyroglutamate were higher and levels of acetate and glucose were lower in both MS and NMOSD. Citrate was lower in MS patients, whereas lactate was higher in only NMOSD specifically. The shared feature of metabolic changes between MS and NMOSD may be related to altered energy metabolism and fatty acid biosynthesis in the brain. Another analysis to characterize relapse and remission status showed that isoleucine and valine were down-regulated in MS relapse compared to MS remission. The other metabolites identified in the disease comparison showed the same alterations regardless of disease activity. These findings would be helpful in understanding the biological background of these diseases, and distinguishing between MS and NMOSD, as well as determining the disease activity.

## Introduction

Multiple sclerosis (MS) is the most common autoimmune demyelinating disease of the central nervous system (CNS) affecting more than two million people around the world [1]. MS is a complicated disease with many different immune cells involved in its pathogenesis; T cells in particularly are the most recognized cell type in CNS lesions [2]. Histopathologically, MS is characterized by inflammation of the CNS, demyelination, and glial scarring. Although the exact cause remains elusive, MS is considered to arise in genetically susceptible individuals with environmental factors influencing disease penetrance [3]. Neuromyelitis optica (NMO), another inflammatory autoimmune disease of the CNS, is characterized by severe and recurrent optic neuritis and longitudinal extensive transverse myelitis [4]. Even though NMO was traditionally considered a variant of MS, it is now considered a distinct disease based on its unique biological features. The discovery of a disease-specific immunoglobulin G antibody (NMO-IgG) that selectively binds aquaporin-4, the most abundant water channel in the CNS [5, 6], has led to an increased understanding of NMO and broadened the clinical and neuroimaging spectrum of NMO [7, 8]. Accordingly, the International Panel for NMO Diagnosis (IPND) proposed new diagnostic criteria to cover the full spectrum of disease and suggested use of the unifying term, NMO spectrum disorder (NMOSD) [9].

Diagnosis of MS and NMOSD is based on observed clinical signs and symptoms, in combination with supporting magnetic resonance imaging (MRI) and laboratory tests [9, 10]. Because both MS and NMOSD have characteristic clinical courses with episodes of relapse and remission and share some features, the discrimination of these two diseases is often challenging, especially in the early stages. Nonetheless, it is essential to make an early, accurate diagnosis as some MS disease modifying therapies appear to worsen NMOSD [11, 12]. Thus, biological characterization of the diseases may be a major concern for in-depth understanding of disease status and predictive test at early stage of MS and NMOSD.

Metabolomics is the systematic study of chemical processes involving metabolites in a biological system. Metabolites represent the end products in a cell, tissue, organ, or organism. Metabolic profiling can provide an immediate indication of the physiology of the biological system being examined [13]. Nuclear magnetic resonance (NMR) spectroscopy-based metabolomics is an important metabolomic tool, having the advantages of being quantitative and highly reproducible. Applications of NMR based metabolomics have increased in recent years and it is now widely used in areas such as toxicology, ecology, and epidemiology. It is a valuable and powerful platform that simultaneously provides insight into find out insights into the pathogenesis and mechanisms of a disease [14–18].

Although several studies have characterized the metabolites in the serum [19–23], CSF [20, 24–28] and urine [29] from MS patients, to date a comparative analysis between relapse and remission episodes have not been performed. Because of the heterogeneity of MS disease as well as chemometric and technical limitations, the previously reported metabolic profiles were inconsistent. In the present study, we aimed to identify similar and different metabolic features between MS and NMOSD and to identify those metabolites that might characterize disease activity (relapse and remission) using an NMR based metabolomics approach. We compared the metabolite profile of CSF specimens obtained from healthy controls (HCs), MS, and NMOSD patients. The statistical analysis of variables from NMR spectra provided several key disease-specific and disease activity-specific metabolites.

## Materials and methods

### Patients and CSF samples

CSF samples from 50 patients with MS and 57 patients with NMOSD from the National Cancer Center were analyzed. Control CSF samples were obtained from 17 HCs who underwent lumbar puncture to rule out meningitis. Because the CSF sampling of healthy controls by lumbar puncture is not easy process, the structure of the cohorts have unbalanced classes. The demographic and clinical data of these patients, including gender, age, dates of sampling, and Expanded Disability Status Scale (EDSS) score, were collected retrospectively with information regarding disease status (relapse/remission). Diagnoses of MS or NMOSD were based on the 2010 McDonald criteria and the 2015 International Panel for NMO diagnosis (IPND) criteria, respectively [9, 10]. Demographic and clinical characteristics are summarized in Table 1. This study was approved by the research ethics committee of the National Cancer Center. All the procedures were carried out in accordance with the Institutional Review Board of National Cancer Center (NCC2014-0416), and written informed consent was obtained from all subjects.

**Table 1:** Demographic and clinical characteristics of the patients.

	control	MS	NMOSD
Number of patients	17	50	57
Female to male (n)	13: 4	33: 17	51: 6
Age of onset mean / SD^*^ (range)	NA^**^	30.20 / 8.06 (14–48)	31.49 / 13. 51 (6–64)
Age at sampling mean / SD (range)	33.35 / 8.36 (22–49)	36.10 / 11.56 (14–50)	35.64 / 11.50 (10–65)
EDSS^***^ Median (range)	NA	2.0 (0–8.0)	3.5 (0–9.0)
Activity of disease (n)	NA	Relapse (20) Remission (30)	Relapse (36) Remission (21)

^*^SD, standard deviation

^**^NA, not applicable

^***^EDSS, Expanded disability status scale

### NMR spectra acquisition and processing

CSF specimens were stored at -80 ^o^C until the NMR experiment. Then, 400 μl of CSF sample from HCs and patients was mixed with 95 μl stock solution of NMR buffer containing 580 mM sodium phosphate buffer. The final NMR samples contained 100 mM sodium phosphate buffer (pH 7.0), 2 mM of trimethylsilyl-propanoic acid (TSP) and 10% D_2_O. To identify metabolites, one-dimensional ^1^H-NOESY NMR pulse sequence (noesygppr1d) was acquired at 298 K on a Bruker ASCEND III 600 spectrometer equipped with a cryoprobe. The NOESY pulse sequence was generated with presaturation to suppress the residual water signal. ^1^H-NMR spectrum for each sample consisted of 256 scans with following parameters: spectral width = 12019.2 Hz, spectral size = 65536 points, pulse width (90) = 13.0 μs, relaxation delay (RD) = 5.0 s and a mixing time of 10 ms.

For quantitative metabolomics profiling of CSF samples, spectra were processed with Bruker topspin 3.1 (Bruker GmbH, Karlsruhe, Germany) and Chenomx NMR suite 7.7 (Chenomx Inc., Edmonton, Canada). The identified metabolites were evaluated in ^1^H-^13^C HSQC and 2D ^1^H-TOCSY spectra. Each free induction decay (FID) was zero-filled to 64,000 points and transformed with line broadening (LB) = 0.3 Hz. NMR spectra were manually phased by Bruker topspin 3.1 and baseline corrected using Chenomx NMR suite 7.7 and referenced to TSP at 0.0 ppm. Briefly, the baseline model was built in each spectrum using the algorithm of multipoint baseline correction.

### Statistical analysis and metabolite identification

Thirty-two metabolites were identified using the database stored in Chenomx NMR suite 7.7 and were quantified from the comparison of the internal standard (TSP).

Statistical analysis was performed using the web server-based program, the MetaboAnalyst (v3.0) for metabolic analysis and interpretation [30, 31]. Additionally, we used the SPSS version 23 (IBM). The multivariate analysis was performed as follows. The spectra were classified into three groups: HCs, MS, and NMOSD patients who were clinically diagnosed. The quantified metabolites table was pareto-scaled to reduce the influence of quantity variability among the samples [32].

The data were first analyzed by principal component analysis (PCA) to lower the dimensionality of the data and to acquire an overview by presenting trends, groupings, and potential outliers within the data sets. Samples were considered as outliers when they were situated outside the 95% confidence ellipse region of the model [33]. In addition, orthogonal partial least squares discriminant analysis (OPLS-DA) was applied to characterize the group difference. OPLS-DA maximizes class separation by removing variability irrelevant to class separation and builds a model detecting potential variables involved in discriminating between classes [34, 35]. Significant features were identified based on S-plot. The S-plot from the OPLS-DA model combines the covariance p[1] against the correlation p(corr)[1] loading profiles. This corresponds to combining the contribution or magnitude (modeled covariation) with the effect and reliability (modeled correlation) for the model variables with respect to model component scores, respectively [36]. Corresponding Mahalanobis *p-*values for OPLS-DA score plots were calculated with PCA/PLS-DA utilities [37] to determine the statistical significance of group separation in the OPLS-DA score plots. An observed *p-*value of 0.05 was used to identify statistically significant group separation. The OPLS-DA models were further characterized by their *p-*values obtained from CV-ANOVA (Analysis Of Variance testing of Cross-Validated predictive residuals) [38]. The CV-ANOVA implemented in SIMCA 14.1 (Umetrics AB, Umea, Sweden) was used to assess the reliability of the obtained models. The quality of the OPLS-DA models was estimated by R2 (goodness-of-fit) and Q2 (ability-of prediction) parameters. The 1,000-random permutation test was also performed to validate the quality of the model [39].

The univariate analysis was performed to identify metabolites contributing to the discrimination among groups. The normality and equality of variance of the quantified concentrations of metabolites were evaluated using SPSS. All variables did not satisfy the normality (based on the Kolmogorov-Smirnov test) and equality of variance (based on the Levene’s test). Thus, the non-parametric Kruskal-Wallis test was adopted for this purpose. The multiple comparison between groups was adjusted with Bonferroni’s correction [40]. For the multiple testing correction, acquired *p*-values were adjusted using Benjamini and Hochberg False Discovery Rate (FDR) [41].

To characterize the disease activity in relapse and remission, each disease group (MS and NMOSD) were divided into two groups based on disease activity as follows: relapse group and remission group. For this analysis, we excluded four and five samples with steroid-treated relapse in MS and NMOSD patients, respectively. Because steroid treatment can extensively affect the metabolic status of patients, the metabolic characterization of relapse status can be interfered without omission of these samples. The statistical analysis was performed by following the process described above.

Biomarker analyses for multiple biomarkers based on the Receiver Operating Characteristic (ROC) curve were performed. ROC analysis was utilized to evaluate the values of different metabolites for disease discrimination by assessing the area under the ROC curve (AUC), sensitivity, and specificity [42]. The algorithms for ROC curve calculation of multivariate biomarker were based on OPLS-DA models. ROC curves were generated using 7-fold internal cross validated predicted y-values from OPLS-DA model in the SIMCA program (Ver. 14.1). It is important to find the most appropriate combination of metabolites which can produce an effective prediction power. In OPLS-DA model, potential biomarker candidates were selected based on values of variable importance in project (VIP) of all variables. The VIP value of each variable in the model was calculated to indicate its contribution the separation. A higher VIP value represents a stronger contribution to classification between groups [43]. The optimal number of metabolites was obtained based on the AUC values.

## Results

### Metabolic characteristics of MS and NMOSD: Multivariate modeling and univariate analysis

A ^1^H NMR spectrum was acquired for each of the 124 CSF samples collected from 17 HCs, 50 (Relapse 20 and Remission 30) MS patients and 57 NMOSD patients (Relapse 36 and Remission 21) (Table 1).

A total of 32 metabolites identified from the CSF analyses were shown in S1 Table, with a mean concentration and standard deviation. PCA was first applied to each group of samples to explore the basic group differentiation. The PCA scatter plot among the two principal components covers 81.8% of the quantified metabolites data. PCA analysis showed eight samples (three from MS patients and five from NMOSD patients) lay outside the borderline of the 95% confidence ellipse (S1 Fig). Thus, these samples were excluded for further statistical analysis.

**Fig 1:**
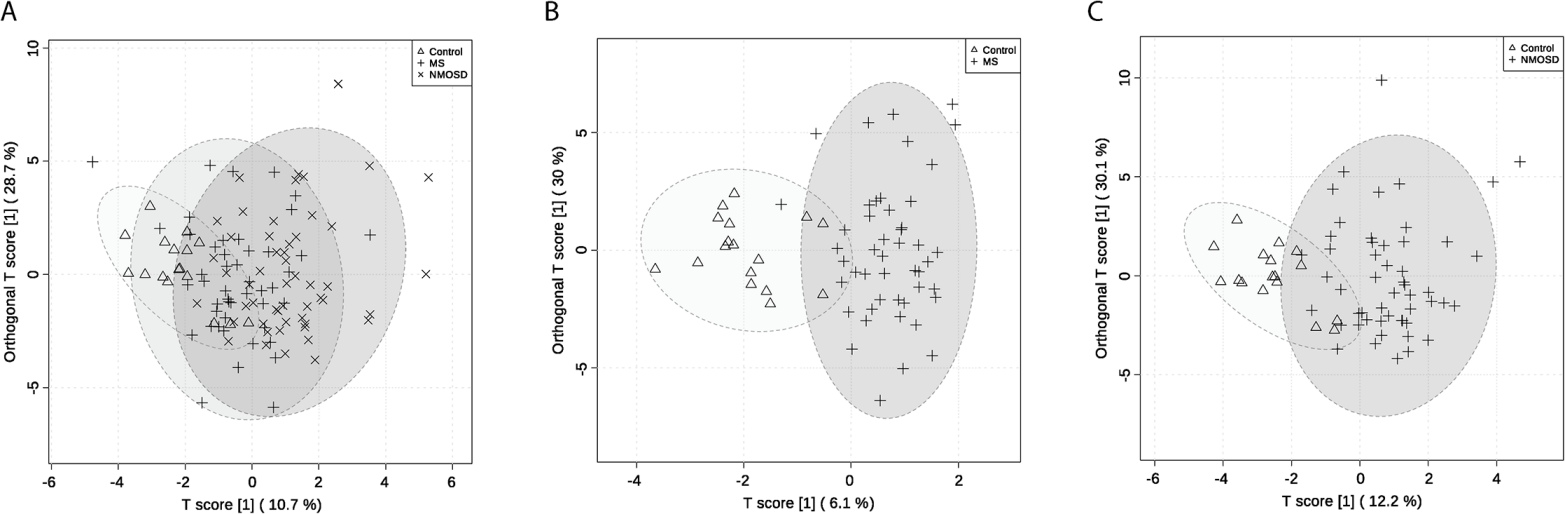
OPLS-DA models for group separation. OPLS-DA scores plots are shown. The triangles in the score plot represents the control sample, the cross signs represent MS patient and the multiplication signs represent NMOSD patients (A). The OPLS-DA models of two groups comparison are shown in B and C. The triangles in the score plot represent the control sample, the cross signs represent MS and NMOSD patient, respectively. The 95% confidence ellipse of the group is depicted in light gray. The created OPLS-DA models (A, B, and C) showed the *p*-values of CV-ANOVA were lower than 0.01 (2.408e-4, 1.544e-7, and 3.759e-7, respectively). The permutation tests for all models showed the empirical *p*-values of R2Y and Q2 were below 0.001. The Mahalanobis *p*-value between two groups (HCs–MS patients, HCs–NMOSD patients and MS–NMOSD) in OPLS-DA score plot for three groups were 1.016e-5, 7.388e-8 and 2.652e-7, respectively (A). The Mahalanobis *p-*values for two group comparison were 1.600e-10 and 4.957e-12, respectively (B and C).

OPLS-DA was employed as a supervised statistical method to clarify the discrimination among the three groups. Fig 1A shows the 2D score plot of OPLS-DA of the three groups. The result showed a moderate separation of three groups along the components with predictive abilities (R2 = 0.443, and Q2 = 0.234, S2 Fig). The significance of the model was validated by the CV-ANOVA (*p*-value = 2.408e-4). The 1,000 permutation tests showed that both the empirical *p*-values of R2Y and Q2 were below 0.001 (0/1000). The Mahalanobis *p*-value between two groups (HCs–MS patients, HCs–NMOSD patients and MS–NMOSD) in OPLS-DA score plot for three groups were 1.016e-5, 7.388e-8 and 2.652e-7, respectively. The margin of the MS group was more overlapped with both the control and NMOSD groups. This may imply that the overall metabolic characteristic of MS patient CSF is more diverse than that of NMOSD patients. The OPLS-DA model between two groups (HCs–MS patients) showed improved separation: the *p-*value calculated from the Mahalanobis distances was less than 0.05 (1.600e-10) and the R2 value was 0.738 and the Q2 value was 0.408 (Fig 1B). The *p*-value of CV-ANOVA was below 0.01 (*p* = 1.544e-7). The permutation test for the OPLS-DA model of the two groups showed the observed R2 and Q2 values were higher than those of the permuted model (*p*<0.001, S3 Fig), indicating the model was regarded predictable one. As for NMOSD, the group separation between the control and the NMOSD group was also improved with the *p-*values obtained from the Mahalanobis distances (4.957e-12) and satisfactory predictive abilities (R2 = 0.589, Q2 = 0.406, *p*-value of CV-ANOVA = 3.759e-7) (Fig 1C). All observed R2 and Q2 values of the OPLS-DA model for HCs and NMOSD patients were higher than those of the permuted test, revealing predictability and goodness of fit (S3 Fig).

**Fig 2:**
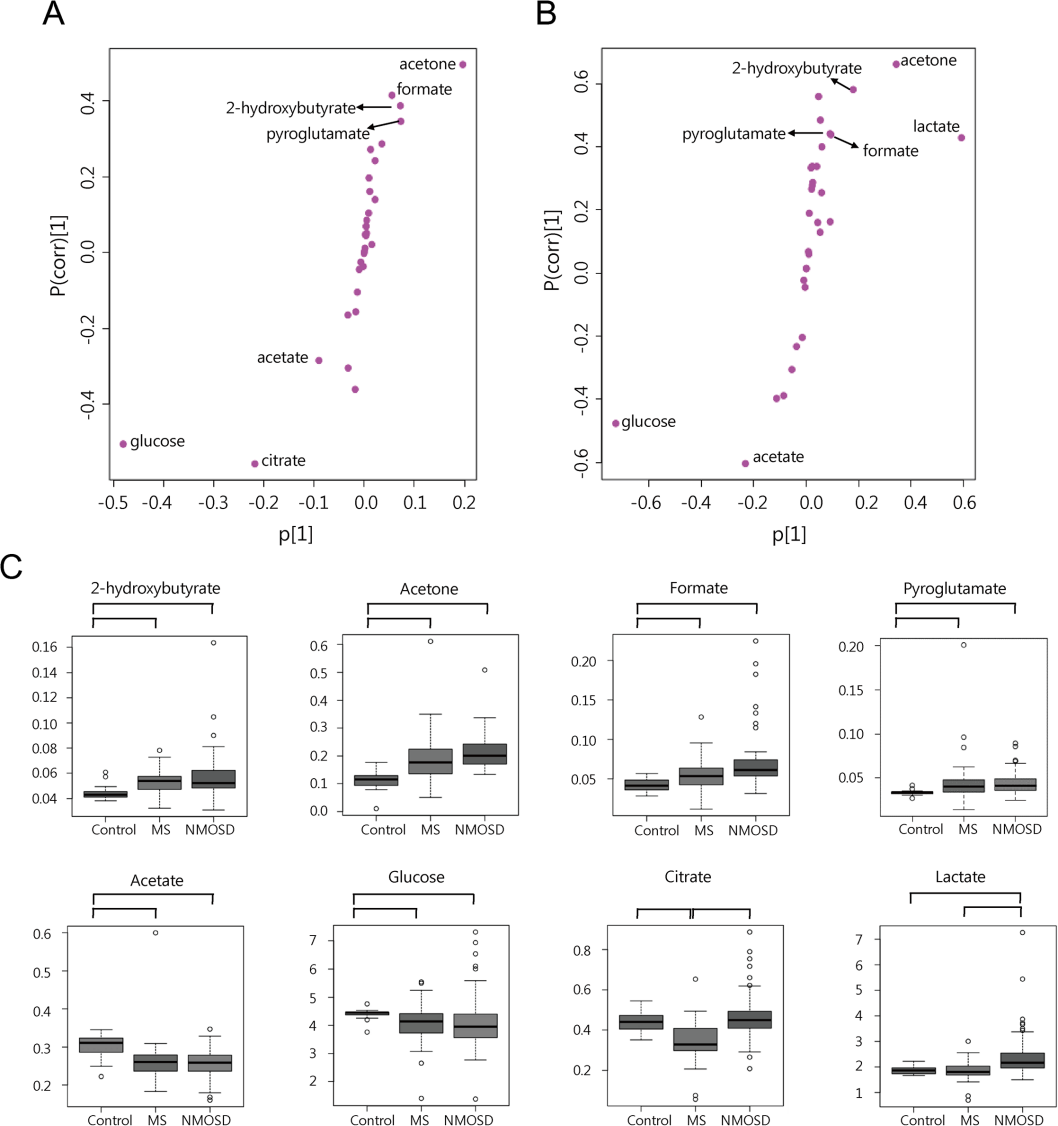
**The S-plot of OPLS-DA models between two groups of HCs-MS patients (A) and HCs-NMOSD patients (B). Eight metabolites with significant difference between the control and patients groups (C).** The S-plot between two groups of HCs-MS patients (A) and HCs-NMOSD patients (B) from OPLS-DA are shown and metabolites that were highly contributed to the group separation are depicted on the plots. The triangles represent the control sample and the cross signs represent patient group. The important metabolites (*p* < 0.05, FDR <0.05) with the strongest association to disease are depicted on the S-plot. Box and whisker plots of eight metabolites using quantified concentrations are illustrated (C). The scale of concentration is millimolar (mM). Eight metabolites including 2-hydroxybutyrate, acetone, formate, pyroglutamate, acetate, glucose, citrate, and lactate showed significant changes. The groups of which the comparison was identified as significant are linked with lines. The horizontal line in the middle portion of the box is median value. The bottom and top boundaries of boxes represent lower and upper quartile. The open circles represent outliers.

The univariate analysis was performed to identify metabolites contributing to the discrimination of the groups. We used the non-parametric Kruskal-Wallis test, followed by Bonferroni’s correction for multiple comparison among the three groups. The adjusted *p*-values were corrected by the multiple testing correction, Benjamini and Hochberg FDR. The final *p-*value that is smaller than 0.05 was considered significant (Table 2). As a result, 2-hydroxybutyrate, acetone, formate and pyroglutamate were up-regulated in MS and NMOSD groups while glucose and acetate were down-regulated in MS and NMOSD compared with HCs. Interestingly, two metabolites were changed either in MS patients or in NMOSD patients. Citrate was lower only in MS patients and lactate and were higher only in NMOSD patients. The S-plot from OPLS-DA models (Fig 1B and 1C) also revealed that the eight metabolites that are responsible for the observed separation (Fig 2A and 2B). The box and whisker plots of metabolites using the quantified concentration are shown in Fig 2C. The representative ^1^H NMR spectra obtained from the CSF samples of HCs, MS patients and NMOSD patients are shown in S4A–S4C Fig.

**Table 2:** Metabolites with significant difference between the control and patients groups.

Metabolite	Multiple comparison (adjusted *p*-value^*^)	FDR^***^	Metabolic change^****^		Mean(SD) of group (mM)		
			M	N	C	M	N
2-hydroxybutyrate	^**^C-M(0.003) C-N(<0.001)	<0.05 <0.05	Δ	Δ	0.0420 (0.0086)	0.0544 (0.0204)	0.0718 (0.0373)
Acetone	C-M(0.001) C-N(<0.001)	<0.05 <0.05	Δ	Δ	0.1112 (0.0364)	0.1868 (0.0864)	0.2135 (0.0627)
Formate	C-M(0.003) C-N(0.001)	<0.05 <0.05	Δ	Δ	0.0449 (0.0061)	0.0530 (0.0101)	0.0576 (0.0197)
Pyroglutamate	C-M(0.001) C-N(<0.001)	<0.05 <0.05	Δ	Δ	0.0332 (0.0030)	0.0454 (0.0265)	0.0443 (0.0135)
Acetate	C-M(0.001) C-N(<0.001)	<0.05 <0.05	∇	∇	0.2993 (0.0033)	0.2626 (0.0565)	0.2563 (0.0396)
Glucose	C-M(0.001) C-N(<0.001)	<0.05 <0.05	∇	∇	4.5681 (0.2540)	4.0432 (0.6704)	4.1501 (0.9873)
Citrate	C-M(0.003) M-N(<0.001)	<0.05 <0.05	∇	ᅳ	0.4352 (0.0532)	0.3451 (0.0969)	0.4698 (0.1218)
Lactate	C-N(0.002) M-N(<0.001)	0.005 <0.05	ᅳ	Δ	1.8832 (0.1715)	1.8521 (0.3670)	2.4513 (0.9397)

^*^ Adjusted *p*-value was calculated by Bonferroni’s correction.

^**^ C, healthy control; M, MS; N, NMOSD

^***^ FDR was calculated by Benjamini-Hochberg method.

^****^ Compared to the values of healthy controls, Δ indicates increase, ∇ indicates decrease, and ᅳ indicates no significant change.

**Fig 3:**
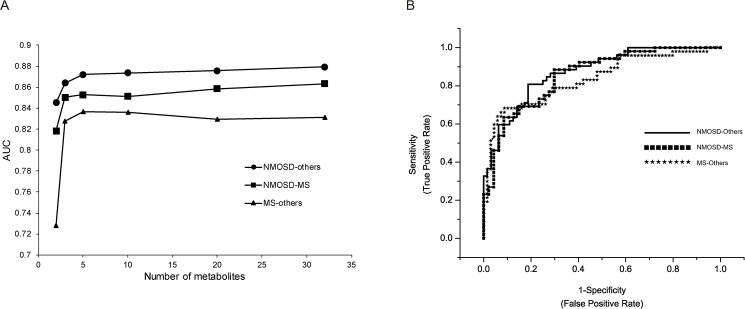
The ROC curve analysis for the composite metabolites. ROC curves of each group comparison were created by 7-fold cross validated predicted y-values from OPLS-DA model. The top 2, 3, 5, 10, 20, 32 (max) important variables based on the VIP values from OPLS-DA model were used to build classification models. The AUC values were obtained from OPLS-DA models of NMOSD-others, NMOSD-MS, and MS-others with combination of metabolites (A). The combination of five metabolites that showed high importance in the group comparison of NMOSD-others, NMOSD-MS, and MS-others provided the AUC value, 0.872, 0.856, and 0.835, respectively (B).

Next, we explored the discriminant candidates of metabolites that separate each disease group (HCs, MS, NMOSD) against each other using ROC curve analysis. For this, several grouping such as MS-others, NMOSD-others, and NMOSD-MS were examined and the grouping of NMOSD-others was found to provide the most discrimination power. The optimal number and composite of biomarkers was determined by monitoring AUC values obtained from the OPLS-DA. The maximum AUC values were saturated around 5 metabolites combination (Fig 3A). The potential biomarker candidates were citrate, lactate, glucose, acetone, and acetate that showed the highest VIP value in the model. The achieved AUCs of NMOSD-others (HCs + MS), MS-NMOSD, and MS-others (HCs + NMOSD) were 0.872, 0.856, and 0.835, respectively (Fig 3B). The result of ROC curve analysis using PLS-DA algorithm showed a slightly lower AUC values than that of OPLS-DA model (S5 Fig). Thus, the model comparing NMOSD and others showed better discrimination power than the model of MS-NMO or MS-others. This result may suggest that the combination use of metabolites can provide a useful tool for the discrimination between MS and NMOSD.

### Metabolic characteristic depending on disease activity in MS and NMOSD patients

The disease activity (relapse and remission) of MS and NMOSD patients was analyzed by comparing metabolic changes between two states. The samples of each disease group were separated into two groups, relapse and remission, based on disease activity. Multivariate analysis such as PCA and OPLS-DA were performed separately for MS and NMOSD (control—MS relapse—MS remission and control—NMOSD relapse—NMOSD remission). PCA analysis did not show clear separation between groups in both case of MS and NMOSD (S5 Fig). In PCA score plot, two (1 remission sample, 1 relapse sample in MS) and five (2 remission samples, 3 relapse samples in NMOSD) outliers were detected, respectively. OPLS-DA was used to clarify the discrimination among the three groups containing 61 samples (control—MS relapse—MS remission) and 64 samples (control—NMOSD relapse—NMOSD remission), respectively. We excluded four and five samples with steroid-treated relapse in MS and NMOSD patients respectively, to escape potential effect of steroid treatment on the metabolic changes.

The univariate analysis showed notable changes of several metabolites depending on the disease states. The statistical analysis was performed using the quantified concentration of the changed metabolites (Tables 3 and 4). As a result, 2-hydroxybutyrate, acetone, and formate were higher and acetate and glucose were lower in both relapse and remission of MS and NMOSD. The level of pyroglutamate may tend to be higher in both relapse and remission of MS and NMOSD, even though statistical value was not satisfactory in the case of MS relapse and remission (*p*-values <0.05, FDR values > 0.05). In addition, citrate was lower in both relapse and remission episodes of MS and lactate was higher in both relapse and remission episodes of NMOSD. These results were consistent with the former comparative analysis between control and diseases. Notably, there were disease activity-specific changes in two metabolites. Isoleucine and valine were down-regulated and in MS relapse compared to MS remission. In the case of NMOSD, isobutyrate might be down-regulated in NMOSD relapse compared to remission, since statistical requirement was partially satisfied (*p*-value = 0.002, FDR = 0.064).

**Table 3:** Metabolites with significant difference between the control and the disease activity of MS.

Metabolite	Multiple comparison (adjusted *p-*value^*^)	FDR^***^	Metabolic change^****^		Mean (SD) of group (mM)		
			RL	RM	C	RL	RM
2-hydroxybutyrate	^**^RL-C(0.003) RM-C(0.002)	0.019 <0.05	Δ	Δ	0.0420 (0.0086)	0.0482 (0.0260)	0.0561 (0.0165)
Acetone	RL-C(<0.001) RM-C(0.001)	<0.05 <0.05	Δ	Δ	0.1112 (0.0364)	0.1917 (0.1289)	0.1844 (0.0599)
Formate	RL-C(<0.001) RM-C(0.002)	<0.05 <0.05	Δ	Δ	0.0449 (0.0061)	0.0554 (0.0088)	0.0561 (0.0087)
Acetate	C-RL(<0.001) C-RM(0.001)	<0.05 <0.05	∇	∇	0.2994 (0.0331)	0.2376 (0.0282)	0.2748 (0.0659)
Glucose	C-RL(<0.001) C-RM(0.007)	<0.05 0.045	∇	∇	4.5681 (0.2540)	3.7715 (0.8975)	4.1910 (0.4755)
Citrate	C-RL(<0.001) C-RM(0.004)	<0.05 0.03	∇	∇	0.4352 (0.0532)	0.2973 (0.1136)	0.3592 (0.0654)
Isoleucine	RM-RL (0.003)	<0.05	↓^*****^		0.0079 (0.0013)	0.0067 (0.0028)	0.0092 (0.0027)
Valine	RM-RL (0.003)	<0.05	↓		0.0183 (0.0033)	0.0149 (0.0055)	0.0197 (0.0054)

^*^ Adjusted *p*-value was calculated by Bonferroni’s correction.

^**^ C, healthy control; RL, relapse; RM, Remission

^***^ FDR was calculated by Benjamini-Hochberg method.

^****^ Compared to the values of healthy controls, Δ indicates increase, and ∇ indicates decrease.

^*****^ The level of the metabolite was lowered than that of remission stage.

**Table 4:** Metabolites with significant difference between the control and the disease activity of NMOSD (C, healthy control; RL, relapse; RM, Remission).

Metabolite	Multiple comparison (adjusted *p-*value^*^)	FDR^***^	Metabolic change^****^		Mean (SD) of group(mM)		
			RL	RM	C	RL	RM
2-hydroxybutyrate	^**^RL-C(<0.001) RM-C(<0.001)	<0.05 <0.05	Δ	Δ	0.0420 (0.0086)	0.0757 (0.0437)	0.0697 (0.0303)
Acetone	RL-C(<0.001) RM-C(0.001)	<0.05 <0.05	Δ	Δ	0.1112 (0.0364)	0.2125 (0.0760)	0.2188 (0.0387)
Formate	RL-C(0.003) RM-C(0.004)	0.019 0.018	Δ	Δ	0.0449 (0.0061)	0.0579 (0.0168)	0.0582 (0.0252)
Pyroglutamate	RL-C(0.006) RM-C(<0.001)	<0.05 <0.05	Δ	Δ	0.0332 (0.0030)	0.0426 (0.0142)	0.0437 (0.0080)
Acetate	C-RL(<0.001) C-RM(0.001)	<0.05 <0.05	∇	∇	0.2993 (0.0331)	0.2460 (0.0393)	0.2732 (0.0306)
Glucose	C-RL(0.001) C-RM(0.002)	<0.05 <0.05	∇	∇	4.5681 (0.2540)	3.9866 (0.8970)	4.3001 (1.1814)
Lactate	RL-C(<0.001) RM-C(0.002)	<0.05 <0.05	Δ	Δ	1.8832 (0.1715)	2.5498 (0.8466)	2.3316 (1.1543)
Isoleucine	RL-C(0.006) RM-C(0.002)	<0.05 <0.05	Δ	Δ	0.0079 (0.0013)	0.0112 (0.0056)	0.01188 (0.0062)

^*^ Adjusted *p*-value was calculated by Bonferroni’s correction.

^**^ C, healthy control; RL, relapse; RM, Remission

^***^ FDR was calculated by Benjamini-Hochberg method.

^****^ Compared to the values of healthy controls, Δ indicates increase and ∇ indicates decrease.

The group separation based on OPLS-DA model was also explored for the disease activity of MS and NMOSD. However, all the two group comparison (relapse-remission) and three group comparison (control-relapse-remission) could not yield a plausible model. This may imply that the metabolic feature between the relapse and remission state is not largely different for the current metabolites set, like as the result of univariate statistics described above. Only the OPLS-DA model of control—MS relapse—MS remission showed improved prediction ability (R2 = 0.834, and Q2 = 0.506, S6B Fig) with empirical *p*-values of R2Y: *p* < 0.001 (0/1000) and Q2: *p* < 0.001 (0/1000) in the permutation test. The *p*-value of CV-ANOVA was 3.812e-5 for the model, which supports the reliability of the model. The Mahalanobis *p*-value between two groups (control–MS relapse, control–MS remission and MS relapse–MS remission) in OPLS-DA model for three groups were 1.072e-5, 5.926e-9 and 3.517e-2, respectively, that indicated the statistical significant of group separation. It is noticeable that citrate makes some contribution to the group separation in the model: compared to the level of citrate in the remission state, the level of citrate seems to be a little lower in the relapse state while the statistical difference between them was not significant (S6C Fig, Table 3).

## Discussion

Our multivariate analysis on MS, and NMOSD indicated the overall metabolic characteristics of the diseases. Eight metabolites in MS and NMOSD patients were significantly different from those of healthy people. In both diseases, 2-hydroxybutyrate, acetone, formate and pyroglutamate were up-regulated and acetate and glucose were down-regulated. Two metabolites showed disease-specific changes. Citrate was down-regulated only in MS and lactate was up-regulated only in NMOSD.

**Fig 4:**
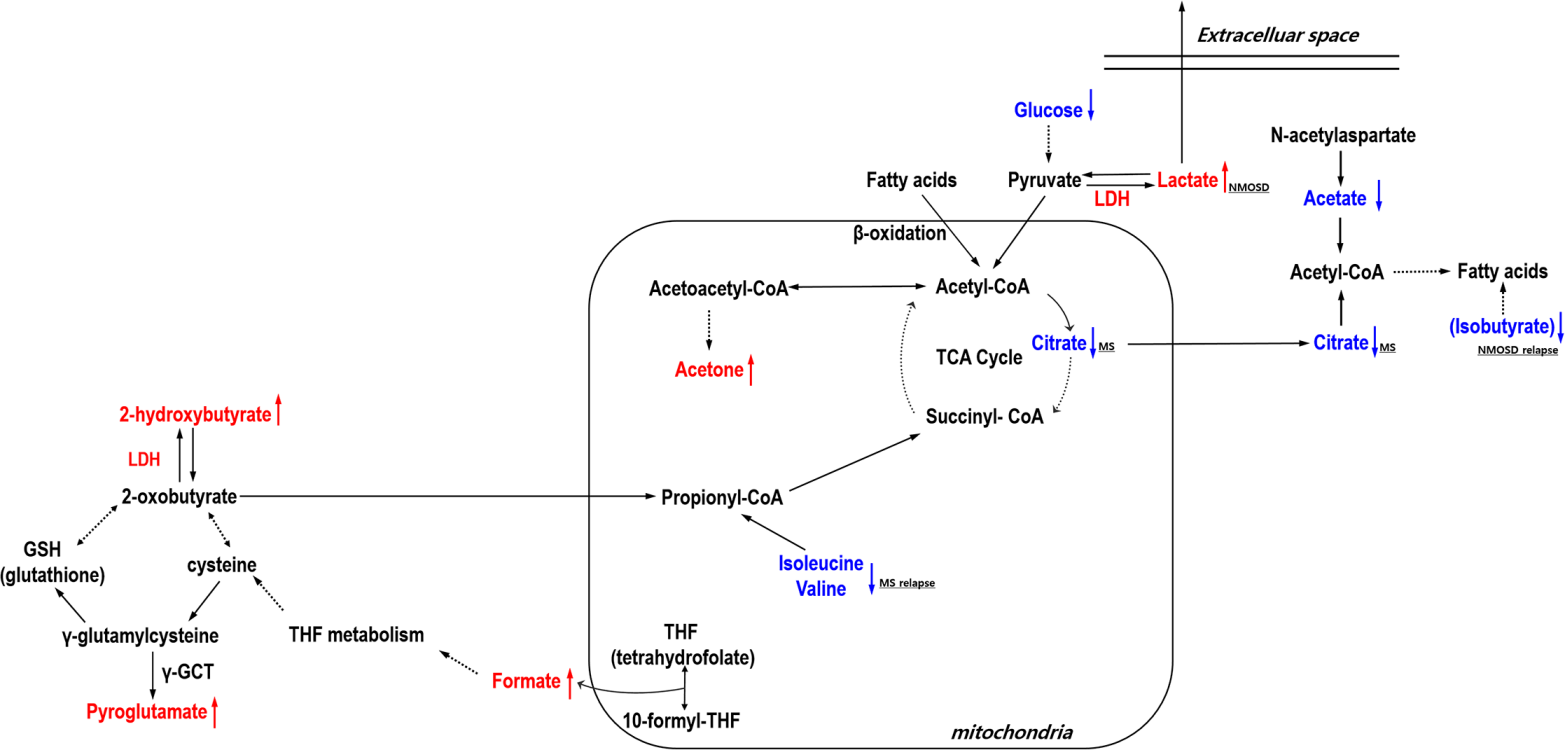
Schematic representation of relevant metabolism involved in MS and NMOSD. The perturbed metabolites in patients are depicted in red and blue. The colored dotted arrows represent up (red)- or down (blue)-regulation of metabolites. The black dotted arrows means not direct but abbreviated pathways. Lower glucose and higher acetone together with lower citrate in MS patients and higher lactate in NMOSD patients may suggest an impaired TCA cycle in mitochondria. Up-regulation of 2-hydroxybutyrate and pyroglutamate in patients indicates that an elevated oxidative stress may be caused by impaired GSH metabolism in neurological diseases such as MS and NMOSD. The elevated level of formate might reflect a malfunction of brain mitochondria as well as oxidative stress. Down-regulation of acetate in the disease groups and down-regulation of citrate in MS patients may indicate a decrease of fatty acid metabolism, particularly in myelin synthesis. γ-GCT: γ-glutamyl cyclotransferase; LDH: lactate dehydrogenase; CoA: coenzyme A.

The shared feature of metabolic changes between MS and NMOSD may be related to altered energy metabolism and fatty acid biosynthesis in the brain (Fig 4). Down regulation of glucose and citrate (in MS) as well as upregulation of lactate (in NMOSD) may support disruption of TCA cycle through pyruvate pathway. Acetone is an end product of ketosis, a metabolic state that produces ketone bodies for use as another fuel for the brain. Both the level of 3-hydroxybutyrate (nonsignificant, *p-*value > 0.05, not shown) and acetone were higher in patients than healthy people in our analysis. The significant increase of acetone may imply that the elevated flux from acetyl-CoA into acetoacetyl-CoA, resulted in production of acetone. Reduced ATP synthesis may ultimately lead to cell death or degeneration, especially as the mitochondria generates most of the energy for neuronal cell [44]. Regarding the myelination of axons, ATP liberated from axons can facilitate myelination by mature oligodendrocytes through a cytokine, leukemia inhibitory factor (LIF) released from astrocytes [45].

It is not clear whether low levels of glucose are caused by their impaired delivery through the blood-brain barrier (BBB) and/or by rapid consumption due to increased energy demand in active MS and NMOSD lesions. Interestingly, the expression level of GLUT1 (SLC2A1), a major glucose transporter in the BBB, is down-regulated in brain lesions of MS patients [46]. Moreover, a recent ^1^H-NMR study reported reduced levels of serum glucose in MS [28]. Consistently, the observations for disturbed energy generation in CNS diseases including MS were reported: mitochondrial dysfunctions detected in MS lesions as well as other neurodegenerative diseases were observed by monitoring mitochondrial gene expression levels [47, 48].

The lactate level in the CSF of NMOSD was relatively elevated [49, 50], which is consistent with the current study results. Lactate is generated by LDH, a large complex consisting of LDHA and LDHB. LDHA produces lactate from pyruvate and LDHB converts lactate into pyruvate to produce energy. In MS patients, the balanced expression of LDHA and LDHB depends on whether the MS lesions is active or inactive [51]. The increase of lactate in the CSF of NMOSD may support the dysfunction of mitochondria because glucose is shunted primarily to lactate through anaerobic glycolysis in the abnormal state of oxidative glucose metabolism [52]. In addition, disruption of the astrocyte-neuron lactate shuttle [53, 54] can also be considered as a cause of unusual level of lactate in NMOSD patients. Glial abnormalities are found in both MS and NMOSD while astrocytes are especially damaged in NMOSD [55], which may be linked to our finding of altered lactate only in NMOSD patients. Previous studies showed the elevation of lactate levels in the CSF were related to disease activity of NMOSD [49, 50], where patients with relapse showed higher lactate levels compared with patients with remission. Our data also showed that lactate levels during of relapse (2.549 ± 0.846 mM) were higher compared with remission (2.332 ± 1.154 mM), although this was not statistically significant.

A high level of 2-hydroxybutyrate was previously suggested to be an early marker for impaired glucose regulation [56]. This may arise due to increased lipid oxidation and oxidative stress because 2-hydroxybutyrate is produced from threonine and methionine catabolism as well as glutathione metabolism. Several studies also suggested the relationship between 2-hydroxybutyrate and neurodegenerative disorders such as dihydrolipoyl dehydrogenase (E3) deficiency [57] and cerebral lactic acidosis [58]. 2-hydroxybutyrate is oxidized to 2-oxobutyrate (alpha-ketobutyrate), which may be transported to the mitochondria and which is prone to oxidative decarboxylation to produce propionyl-CoA, a TCA cycle intermediate [59]. Interestingly, the interconversion between 2-oxobutyrate and 2-hydroxybutyrate is regulated by LDH [60]. Of note, 2-hydroxybutyrate was elevated in the urine of young patients with lactic acidosis [61]. 2-oxobutyrate can be produced by the direct catabolism of threonine or methionine metabolism through homocysteine and cystathionine. Cysteine is incorporated into glutathione (GSH), which is the major component of the non-enzymatic unit of the cellular antioxidant. Oxidative stress is a common pathological characteristics in neurological diseases, including MS [62]. In elevated oxidative stress conditions, the flow of cysteine for the formation of GSH is increased [63]. However, a previous study showed that GSH levels in the CSF of MS patients were significantly lower compared to controls [64]. The activity of antioxidant enzymes such as GSH reductase and glutathione peroxidase in MS patients was shown to be modified [65].

The level of pyroglutamate commonly increased in both disease of MS and NMOSD. Pyroglutamate is another metabolite associated with GSH metabolism. The enzyme glutathione synthetase is important for GSH synthesis from γ-glutamylcysteine. Error in the glutathione synthetase leads to reduced level of GSH and accumulation of γ-glutamylcysteine. The γ-glutamylcysteine synthetase, the first enzyme of the GSH biosynthesis, is under the feedback control of GSH. Decreased GSH levels due to a defect in the glutathione synthetase impair feedback inhibition of γ-glutamylcysteine synthetase enzyme, producing more γ-glutamylcysteine. The γ-glutamyl cyclotransferase (γ-GCT) generates pyroglutamate from γ-glutamylcysteine. This further leads to overproduction of pyroglutamate and to increased pyroglutamate in body fluid including CSF and urinary excretion of pyroglutamate. This condition also leads to severe metabolic acidosis, hemolytic anemia and central nervous system dysfunction [66]. Taken together, the flow of metabolites for GSH pathway may be altered, positively affecting the accumulation of 2-hydroxybutyrate and pyroglutamate. Formate can be linked to the production of GSH through tetrahydrofolate (THF) metabolism and the malfunction of GSH production possibly affects or causes the increase of formate. Thus, the elevated level of formate might also reflect the dysfunction of brain mitochondria as described above. Anyway, high levels of formate is not favorable situation for neuronal cells because it can disrupt the electron transport system of mitochondria and energy generation by inhibiting cytochrome oxidase function, which is the final electron acceptor of the electron transport pathway, thereby leading to a cellular hypoxia.

In terms of fatty acid metabolism, a low level of glucose may provide a metabolic environment in which fatty acid degradation into acetyl-CoA (beta-oxidation) is facilitated. This situation is probably not favorable for the myelination of axons. A low level of citrate in the CSF of MS patients may also provide unfavorable surroundings for the biosynthesis of fatty acids that result in the formation of myelin because acetyl-CoA used for fatty acid synthesis must be shuttled out of the mitochondria into the cytosol in its citrate form (so called citrate shuttle) [67]. In addition, it was reported that the activity of mitochondrial aconitase that catalyzes the interconversion of citrate to isocitrate was higher in MS patients, which may be related to our result showing low citrate levels were only observed in MS patients [68].

Acetate used for myelin lipid biosynthesis in oligodendrocytes is mainly produced from N-acetylaspartate (NAA) degradation by aspartoacylase (ASPA) [69–71]. NAA is a nervous system-specific metabolite and is regarded as an important metabolite in CNS metabolism. The reduced level of NAA in MS patients was reported previously [72–74], which may relate to the reduced level of acetate in our study. Interestingly, in a mouse model of Canavan's disease, a hereditary disorder of CNS development, dysfunction of ASPA reduced brain acetate levels and reduced the biosynthesis of six classes of myelin-associated lipids [75]. This suggests acetate has a critical role in myelin lipid biosynthesis in MS and NMOSD. In addition, low acetate levels may not be advantageous for the normal astrocyte activity of releasing LIF, which is required for myelination of oligodendrocytes described above because astrocytes have a preference for acetate as an energy substrate [76].

We also analyzed the difference between the disease activity (relapse vs. remission) in MS and NMOSD. Metabolites such as 2-hydroxybutyrate, acetone, formate, glucose, acetate, citrate, pyroglutamate, and lactate showed consistent changes between controls and MS and NMOSD patients. However, in MS patients, isoleucine, and valine were down-regulated in relapse stages compared to remission stages. No statistical differences between controls and relapse, and controls and remission, were found for these two metabolites.

The down-regulation of branched chain amino acid (BCAA), isoleucine, and valine, which can be sources for energy production, was also monitored in MS relapse, revealing a change in energy production. Isoleucine and valine can be converted to acetyl-CoA or propionyl-CoA, which are intermediates of the TCA cycle. In addition, reduced BCAAs in MS relapse might influence protein synthesis in the brain and the synthesis of various neurotransmitters. In terms of immunity, low BCAAs may affect the activity of immune cells. It is well known that blood monocytes and T-cells infiltrate into the CNS and are hyperactivated in MS and NMOSD. BCAAs are required for lymphocyte growth and proliferation as well as dendritic cell maturation [77, 78]. In relapse stages where active inflammation occurs, the consumption of BCAAs by lymphocytes in the brain might be increased.

Several studies reported the metabolic changes in the serum [19–22], CSF [20, 24–28] and urine [29] from MS and NMOSD patients. Table 5 and 6 are the summary of the metabolites changes mainly focusing on the eight metabolites that we identified. Metabolic change for three metabolites of 2-hydroxybutyrate, formate and pyroglutamate were uniquely identified in our study. The changes of lactate, acetate, glucose, 3-hydroxybutyrate, phenylalanine, oxaloacetate, and creatinine were very heterogeneous. This inconsistency might come from the heterogeneity of biofluid source as well as chemometric and technical limitations.

**Table 5:** Metabolic profiling in MS.

Metabolite	Metabolic Change	Biofluids for analysis	References
	MS		
2-hydroxybutyrate	Up^*^	CSF	[Our result]
Formate	Up	CSF	[Our result]
pyroglutamate	Up	CSF	[Our result]
Citrate	Down	CSF	[Our result]
	Down	CSF	[24, 27, 28]
	Down	Serum	[20]
	Down	Urine	[29]
Lactate	No change	CSF	[Our result]
	Up	CSF	[25]
	Down	CSF	[28]
	Down	Urine	[29]
Acetate	Down	CSF	[Our result]
	Down	CSF	[24]
	Up	Serum	[20]
	No change	Urine	[29]
Glucose	Down	CSF	[Our result]
	Up	CSF	[24]
	Down	CSF	[28]
	Up	Serum	[20, 21]
Acetone	Up	CSF	[Our result]
	UP	CSF	[28]
	Up	Urine	[29]
3-hydroxybutyrate	Down	CSF	[27]
	Up	CSF	[28]
	Down	Serum	[20]
	Up	Urine	[29]
Phenylalanine	Down	CSF	[27]
	Up	Urine	[29]
Glutamine	Up	CSF	[25]
	Up	Serum	[19]
Myo-inositol	Up	CSF	[27]
Oxaloacetate	Down	Serum	[20]
	Up	Urine	[29]
Creatinine	Up	CSF	[25]
	Down	Serum	[20]
	Up	Serum	[23]
	Down	Urine	[29]

The table lists the metabolites considering their levels and disease in which they are involved, and the references cited in the paper.

^*^ Compared to the values of controls, Up means increase, Down means decrease. Control group of reference [19, 21, 23, 28, 29] are healthy volunteers and control groups in reference [20, 24, 25, 27] have other disease except MS.

**Table 6:** Metabolic profiling in NMOSD.

Metabolite	Metabolic Change	Biofluids for analysis	References
	NMOSD		
2-hydroxybutyrate	Up^*^	CSF	[Our result]
Formate	Up	CSF	[Our result]
pyroglutamate	Up	CSF	[Our result]
Citrate	No change	CSF	[Our result]
	Down	Urine	[29]
Lactate	Up	CSF	[Our result]
	Up	Serum	[19]
	Down	Urine	[29]
Acetate	Down	CSF	[Our result]
	Up	Serum	[19]
	Up	Urine	[29]
Glucose	Down	CSF	[Our result]
Acetone	Up	CSF	[Our result]
	Down	Urine	[29]
3-hydroxybutyrate	Down	Urine	[29]
Phenylalanine	Up	Urine	[29]
Oxaloacetate	Up	Urine	[29]
Creatinine	Down	Urine	[29]

The table lists the metabolites considering their levels and disease in which they are involved, and the references cited in the paper.

^*^ Compared to the values of controls, Up means increase, Down means decrease. Control group of reference [19, 29] are healthy volunteers.

In conclusion, our findings give us an insight to the pathologic mechanism of MS and NMOSD: several metabolite changes related to impaired energy metabolism linked to the TCA cycle in mitochondria (lower glucose and citrate; higher acetone and lactate), imbalanced control for lipid synthesis (lower acetate and citrate) and increased oxidative stress (higher 2-hydroxybutyrate, pyroglutamate, and formate) may be related to the pathogenesis of MS and NMOSD. Even though our study may enhance scientific knowledge of MS and NMOSD, obtaining of plausible multivariate models for discrimination of the disease activity was not successful. This might be related with the limited number of samples and limited number of metabolites that were quantified. In this regard, investigation with large sample size would be helpful for validation of our findings and for discover of the unrevealed features. In addition, the diagnostic model could be improved with incorporation of several biochemical and clinical parameters.

## Supporting information

S1 Fig PCA analysis of control and disease groups. The red circles in the score plot represent the healthy control sample, the green circle represents the MS patients, and the blue circles represent the remission patients. The 95% confidence ellipse of the group is depicted in each color. The first principal component (PC1) accounts for 57.9% of the total variation present in the dataset and the second principal component (PC2) accounts for 23.9% of the total variation. Thus, the PCA scatter plot among the two principal components covers 81.8% of the quantified metabolites data.
(TIF)

S2 Fig The overview of OPLS-DA model for three groups and the permutation test. The OPLS-DA model was constructed by MetaboAnalyst 3.0 and validated with random permutation test. The permutation number was set to be 1,000. Two components model of the OPLS-DA as shown in the above figure. R2 is the correlation index, which refers to the goodness of fit or the explained variation. Q2 refers to the predicted variation or quality of prediction (A). The permutation result of the OPLS-DA model for the three groups showed the empirical *p*-values of R2 (p < 0.001) and Q2 (p < 0.001), which means the null hypothesis is rejected (B).
(TIF)

S3 Fig The overview of OPLS-DA model for two groups and the permutation test. The OPLS-DA model was constructed by MetaboAnalyst 3.0 and validated with random permutation test. The permutation number was set to be 1,000. The permutation result of the OPLS-DA model for the two groups (control-MS (A) and control-NMOSD (B)) showed the empirical *p*-values of R2 (*p* < 0.001) and Q2 (*p* < 0.001), which means the models are valid.
(TIF)

S4 Fig **Three representative** ^**1**^**H NMR spectra from the CSF samples of HCs (A), MS patients (B) and NMOSD patients (C). Overlay of** ^**1**^**H-NMR spectra of HCs (black), MS patients (green) and NMOSD patients (red) (D).** ^1^H NOSEY spectra were processed and manually phased using Bruker Topspin 3.1. Metabolites with significant differences between the control and patients groups such as 2-hydroxybutyrate, acetone, formate, pyroglutamate, acetate, glucose, citrate, and lactate are depicted in the figure (D).
(TIF)

S5 Fig The ROC curve analysis for the composite metabolites. ROC curves of each group comparison were created by MCCV using balanced subsampling. Two thirds (2/3) of the samples in each MCCV are used to evaluate the variable importance. The top 2, 3, 5, 10, 20, 32 (max) important variables were used to build classification models. The PLS-DA algorithm was hired as the classification method with two latent components since the algorithm provided the best performance. The AUC values were obtained from PLS-DA models of NMOSD-others, NMOSD-MS, and MS-others with combination of metabolites (A). The combination of five metabolites that showed high importance in the group comparison of NMOSD-others, NMOSD-MS, and MS-others provided the AUC value, 0.861, 0.829, and 0.771, respectively (B).
(TIF)

S6 Fig PCA score plots based on disease activity of MS and NMOSD. PCA scores plot of MS activity (A) and NMOSD activity (B) are depicted. The red circles in the score plot represent the control sample, the green circles represent the relapse patients, and the blue circles represent the remission patients. The 95% confidence ellipse of the group is depicted in each color. The first principal component (PC1) accounts for 64.4% (A) and 56.7% (B) of the total variation present in the dataset and the second principal component (PC2) accounts for 10.5% (A) and 29% (B) of the total variation. Thus, the PCA scatter plot among the two principal components covers 74.9% (A) and 95.5% (B) of the original data. Two (2 remission sample, 1 relapse sample) and five (2 remission samples, 3 relapse samples) outliers are detected in MS (A) and NMOSD (B) disease activity groups.
(TIF)

S7 Fig An example of OPLS-DA model for disease activity. The OPLS-DA model was constructed and validated with CV-ANOVA and random permutation test. OPLS-DA scores plots of MS activity (A) are depicted. The red circles in (A) represent the control sample, the green circles represent the relapse patients, and the blue circles represent the remission patients. The 95% confidence ellipse of the group is depicted in each color. The Mahalanobis *p*-value between two groups (control–MS relapse, control–MS remission and MS relapse–MS remission) in OPLS-DA model for three groups were 1.072e-5, 5.926e-9 and 3.517e-2, respectively. The *p*-value of CV-ANOVA was 3.811e-5. (B) The permutation number was set to be 1,000. The observed R2 and Q2 values of the OPLS-DA model were higher than those obtained from the permuted tests, revealing predictability and goodness of fit. The permutation result of the OPLS-DA model for the three groups (control-MS relapse-MS remission) showed the empirical *p*-values of R2 (*p* < 0.001) and Q2 (*p* < 0.001). (C) S-plot of the OPLS-DA model. Influence of the model variable is shown. Glucose and citrate were relatively lowered in the relapse state compared to those in the remission state. Two metabolites as well as acetone seem to mainly contribute for group separation.
(TIF)

S1 Table The quantification of each metabolite (unit, mM). Thirty-two metabolites were identified using the database stored in Chenomx NMR suite 7.7 and were quantified from the comparison of the internal standard (TSP). Data are presented as mean and standard deviation (SD).
(DOCX)
