# Supplementary figures and images for: Metabolomic profiling of CSF in multiple sclerosis and neuromyelitis optica spectrum disorder by nuclear magnetic resonance
Source: PLoS One. 2017 Jul 26;12(7):e0181758. doi: 10.1371/journal.pone.0181758 (PMC5528902; doi:10.1371/journal.pone.0181758)

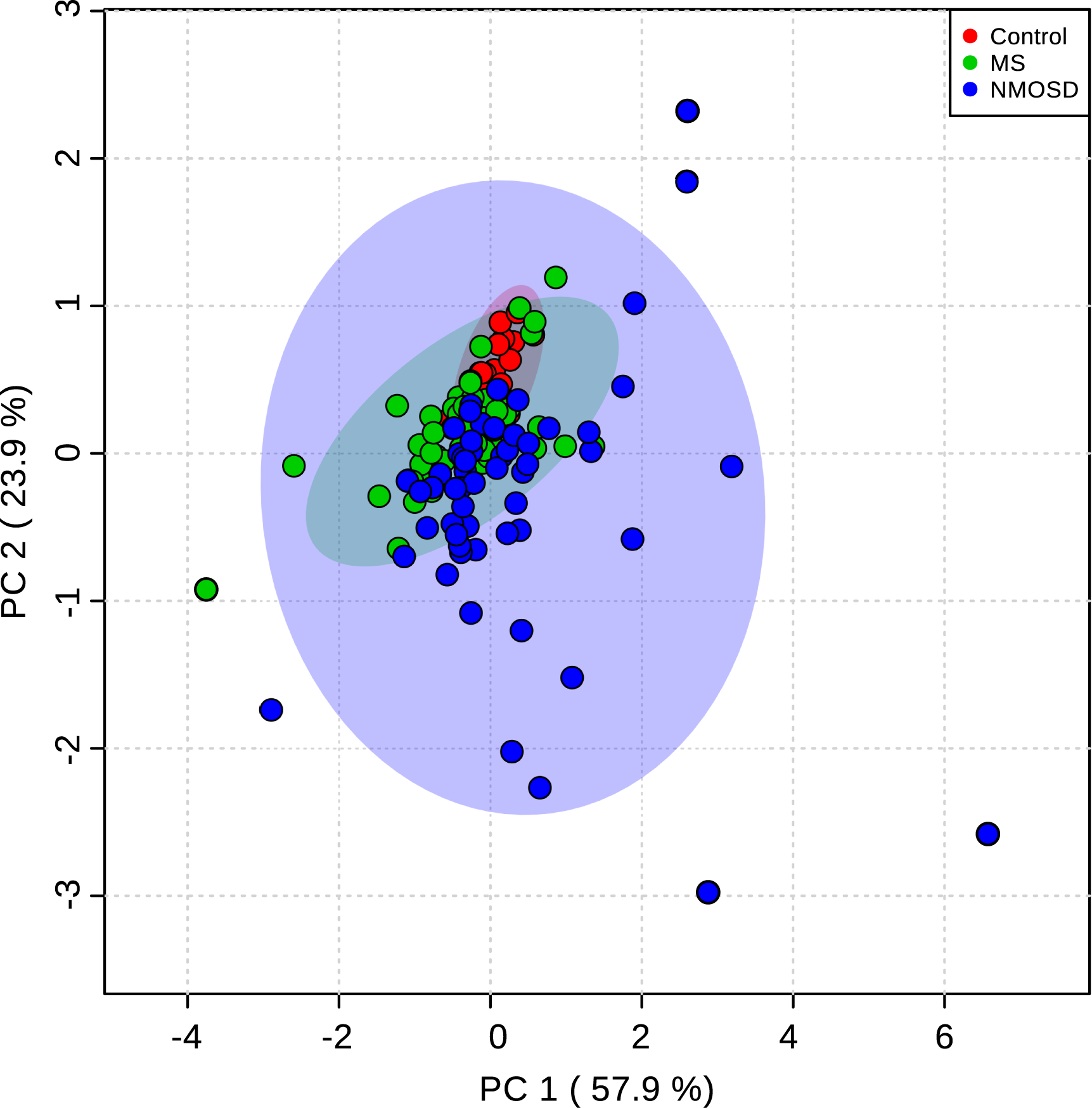

Supplement: S1 Fig — The red circles in the score plot represent the healthy control sample, the green circle represents the MS patients, and the blue circles represent the remission patients. The 95% confidence ellipse of the group is depicted in each color. The first principal component (PC1) accounts for 57.9% of the total variation present in the dataset and the second principal component (PC2) accounts for 23.9% of the total variation. Thus, the PCA scatter plot among the two principal components covers 81.8% of the quantified metabolites data. (TIF) [file pone.0181758.s001.tif]

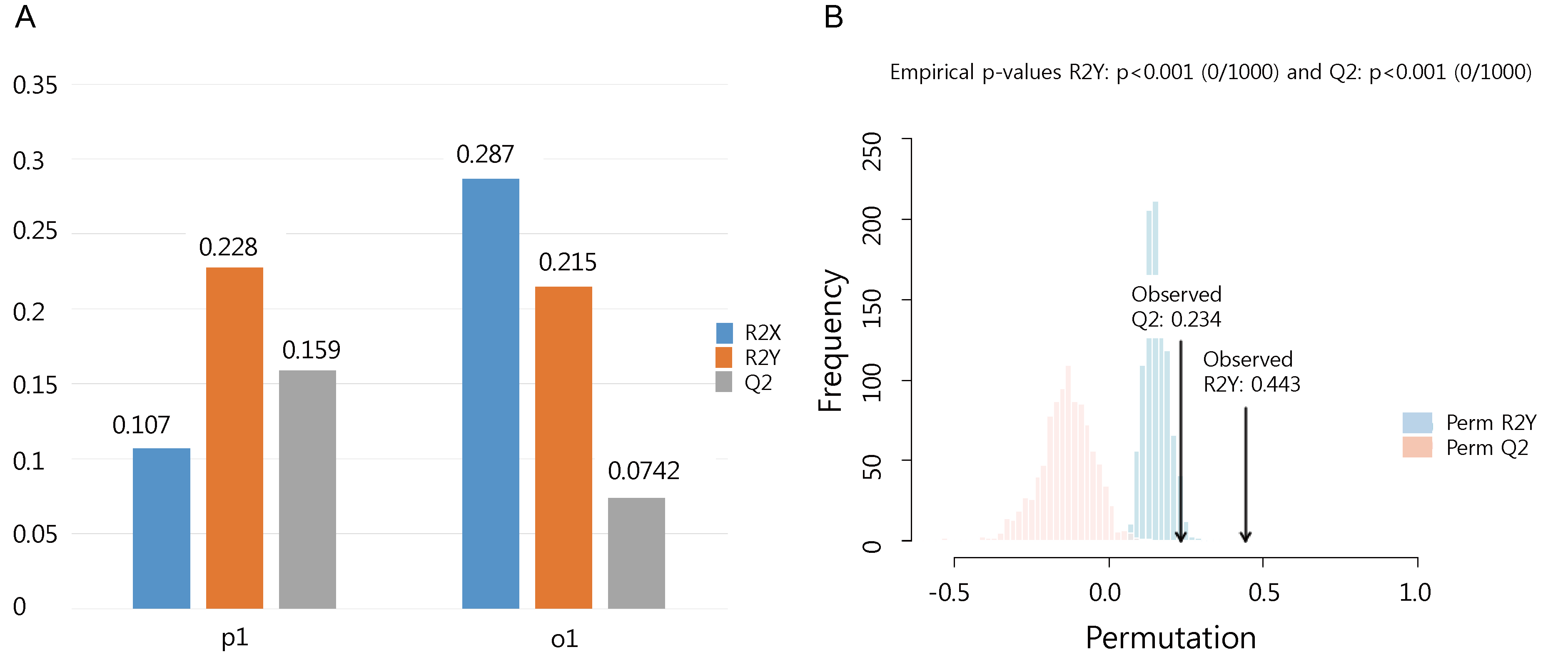

Supplement: S2 Fig — The OPLS-DA model was constructed by MetaboAnalyst 3.0 and validated with random permutation test. The permutation number was set to be 1,000. Two components model of the OPLS-DA as shown in the above figure. R2 is the correlation index, which refers to the goodness of fit or the explained variation. Q2 refers to the predicted variation or quality of prediction (A). The permutation result of the OPLS-DA model for the three groups showed the empirical p-values of R2 (p < 0.001) and Q2 (p < 0.001), which means the null hypothesis is rejected (B). (TIF) [file pone.0181758.s002.tif]

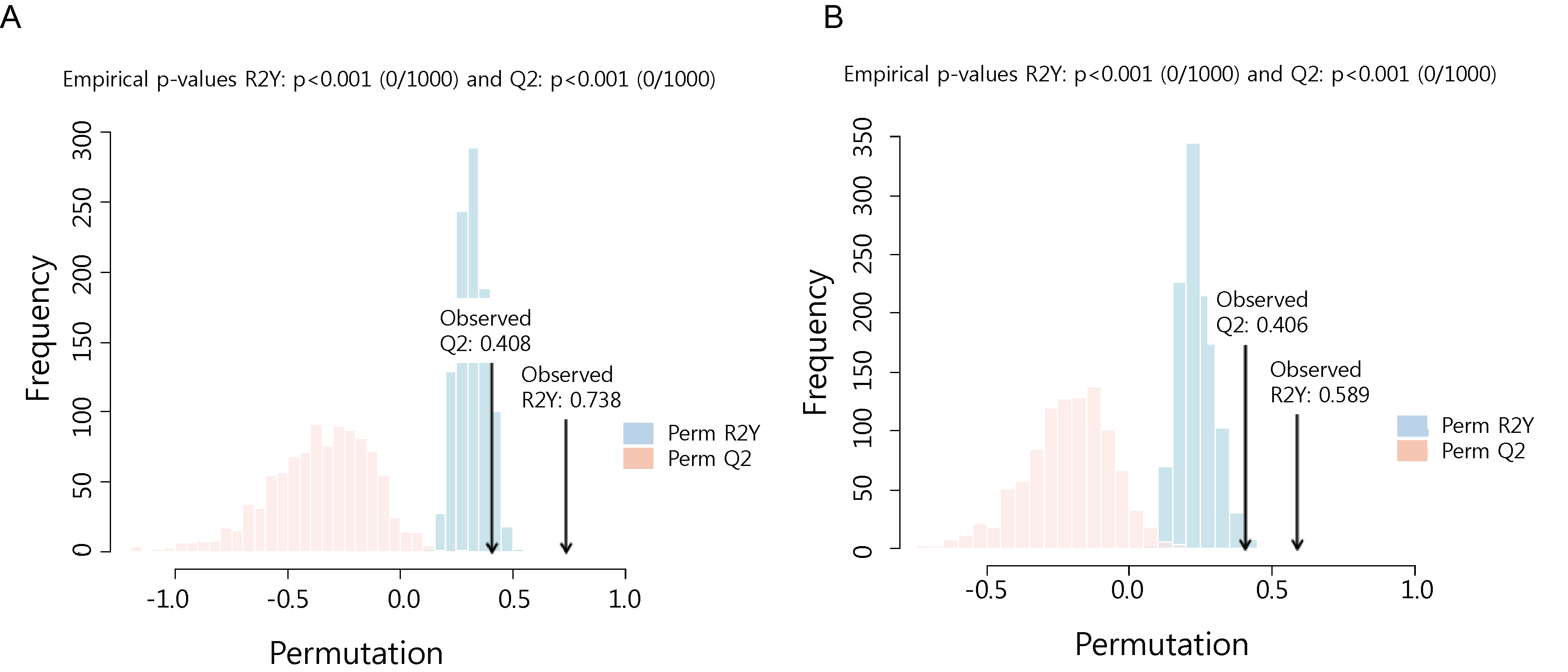

Supplement: S3 Fig — The OPLS-DA model was constructed by MetaboAnalyst 3.0 and validated with random permutation test. The permutation number was set to be 1,000. The permutation result of the OPLS-DA model for the two groups (control-MS (A) and control-NMOSD (B)) showed the empirical p-values of R2 (p < 0.001) and Q2 (p < 0.001), which means the models are valid. (TIF) [file pone.0181758.s003.tif]

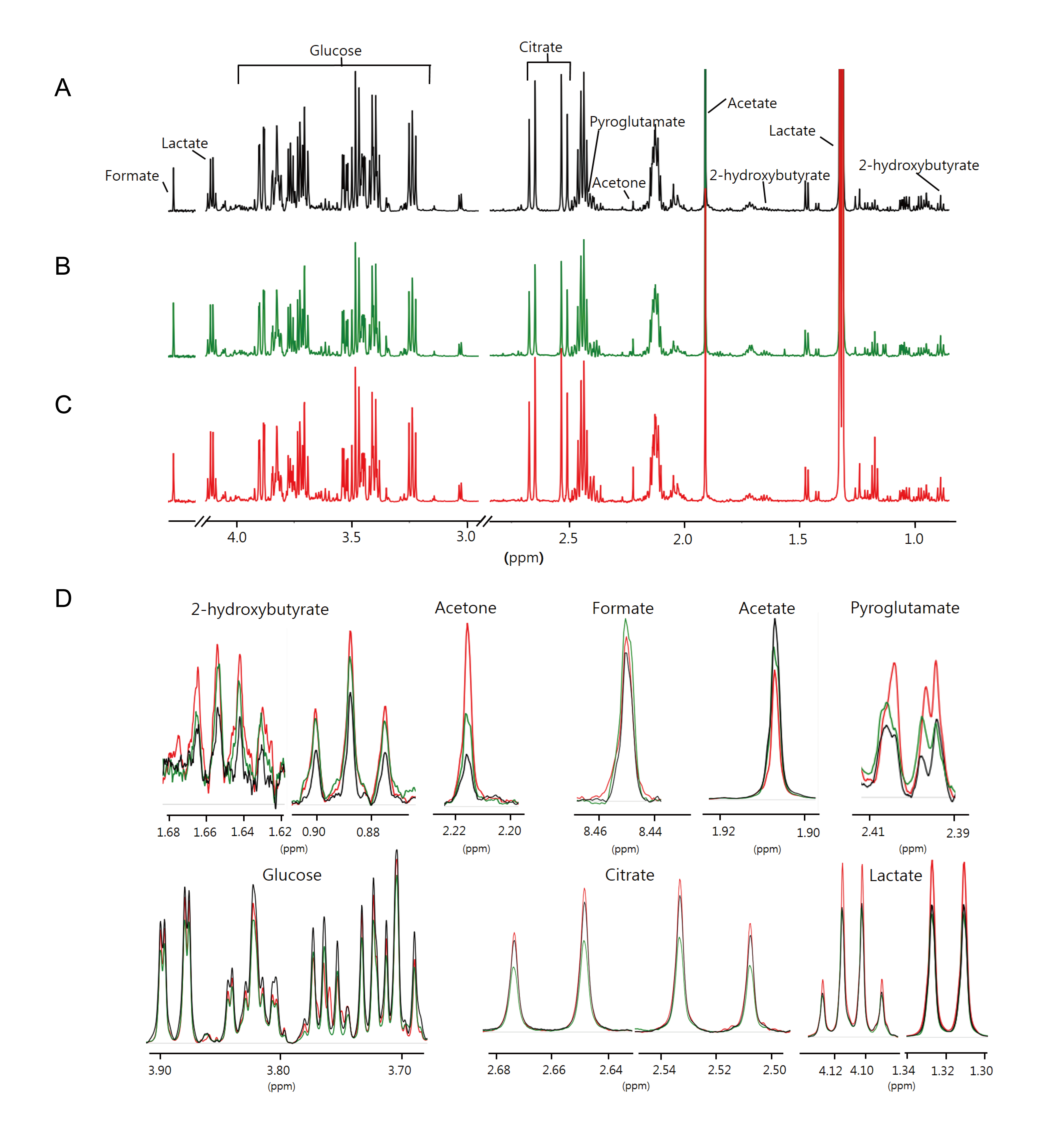

Supplement: S4 Fig — Three representative 1H NMR spectra from the CSF samples of HCs (A), MS patients (B) and NMOSD patients (C). Overlay of 1H-NMR spectra of HCs (black), MS patients (green) and NMOSD patients (red) (D). 1H NOSEY spectra were processed and manually phased using Bruker Topspin 3.1. Metabolites with significant differences between the control and patients groups such as 2-hydroxybutyrate, acetone, formate, pyroglutamate, acetate, glucose, citrate, and lactate are depicted in the figure (D). (TIF) [file pone.0181758.s004.tif]

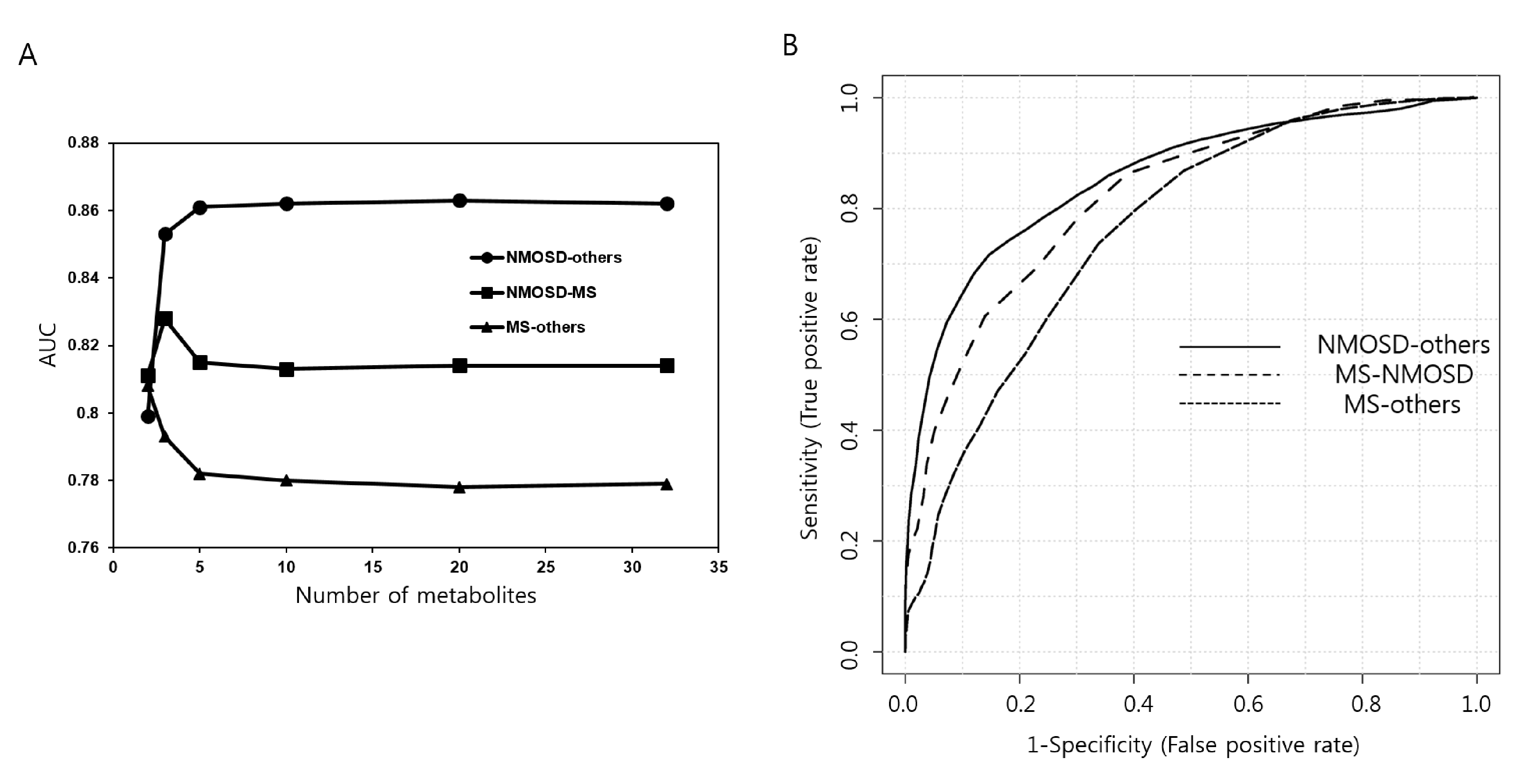

Supplement: S5 Fig — ROC curves of each group comparison were created by MCCV using balanced subsampling. Two thirds (2/3) of the samples in each MCCV are used to evaluate the variable importance. The top 2, 3, 5, 10, 20, 32 (max) important variables were used to build classification models. The PLS-DA algorithm was hired as the classification method with two latent components since the algorithm provided the best performance. The AUC values were obtained from PLS-DA models of NMOSD-others, NMOSD-MS, and MS-others with combination of metabolites (A). The combination of five metabolites that showed high importance in the group comparison of NMOSD-others, NMOSD-MS, and MS-others provided the AUC value, 0.861, 0.829, and 0.771, respectively (B). (TIF) [file pone.0181758.s005.tif]

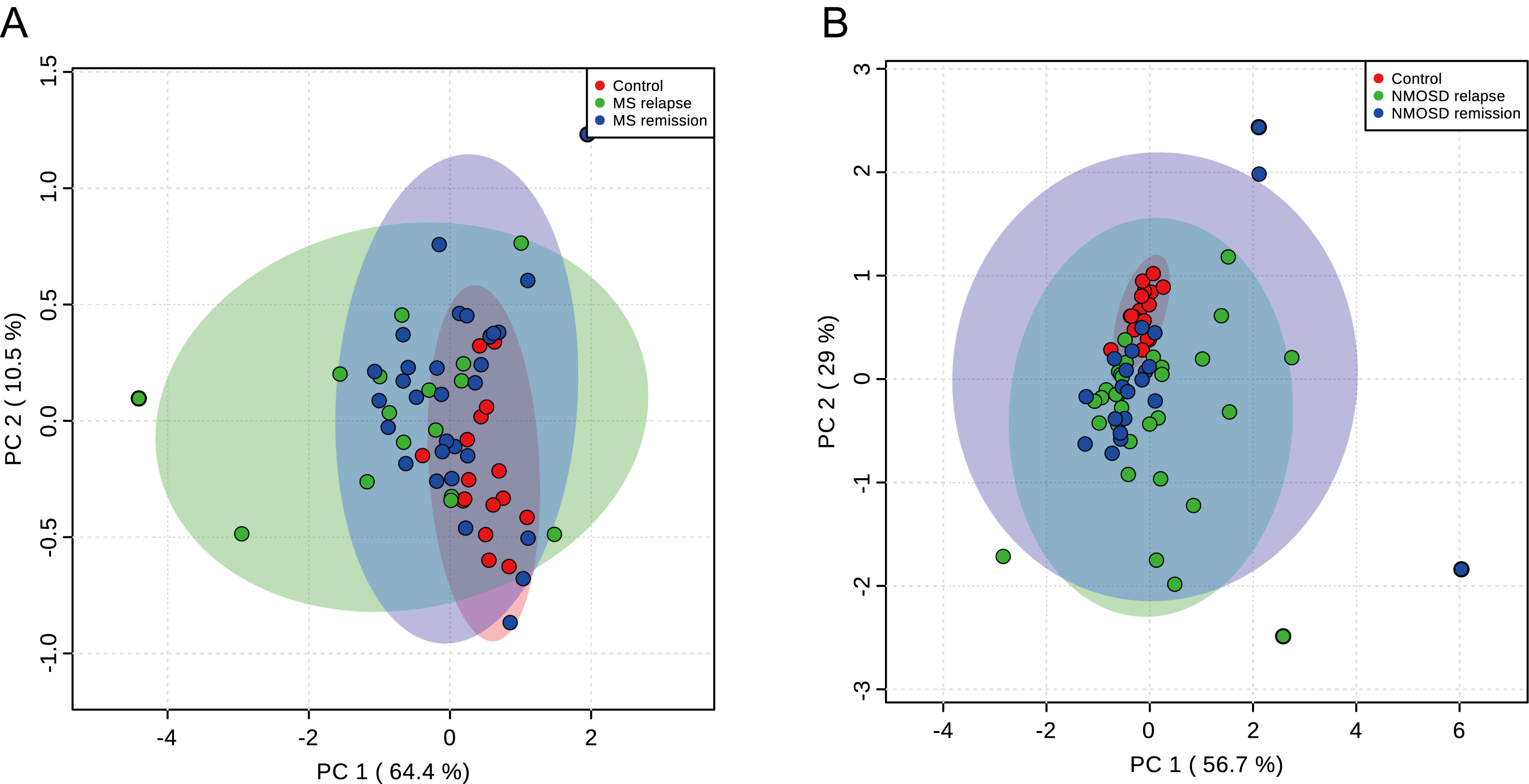

Supplement: S6 Fig — PCA scores plot of MS activity (A) and NMOSD activity (B) are depicted. The red circles in the score plot represent the control sample, the green circles represent the relapse patients, and the blue circles represent the remission patients. The 95% confidence ellipse of the group is depicted in each color. The first principal component (PC1) accounts for 64.4% (A) and 56.7% (B) of the total variation present in the dataset and the second principal component (PC2) accounts for 10.5% (A) and 29% (B) of the total variation. Thus, the PCA scatter plot among the two principal components covers 74.9% (A) and 95.5% (B) of the original data. Two (2 remission sample, 1 relapse sample) and five (2 remission samples, 3 relapse samples) outliers are detected in MS (A) and NMOSD (B) disease activity groups. (TIF) [file pone.0181758.s006.tif]

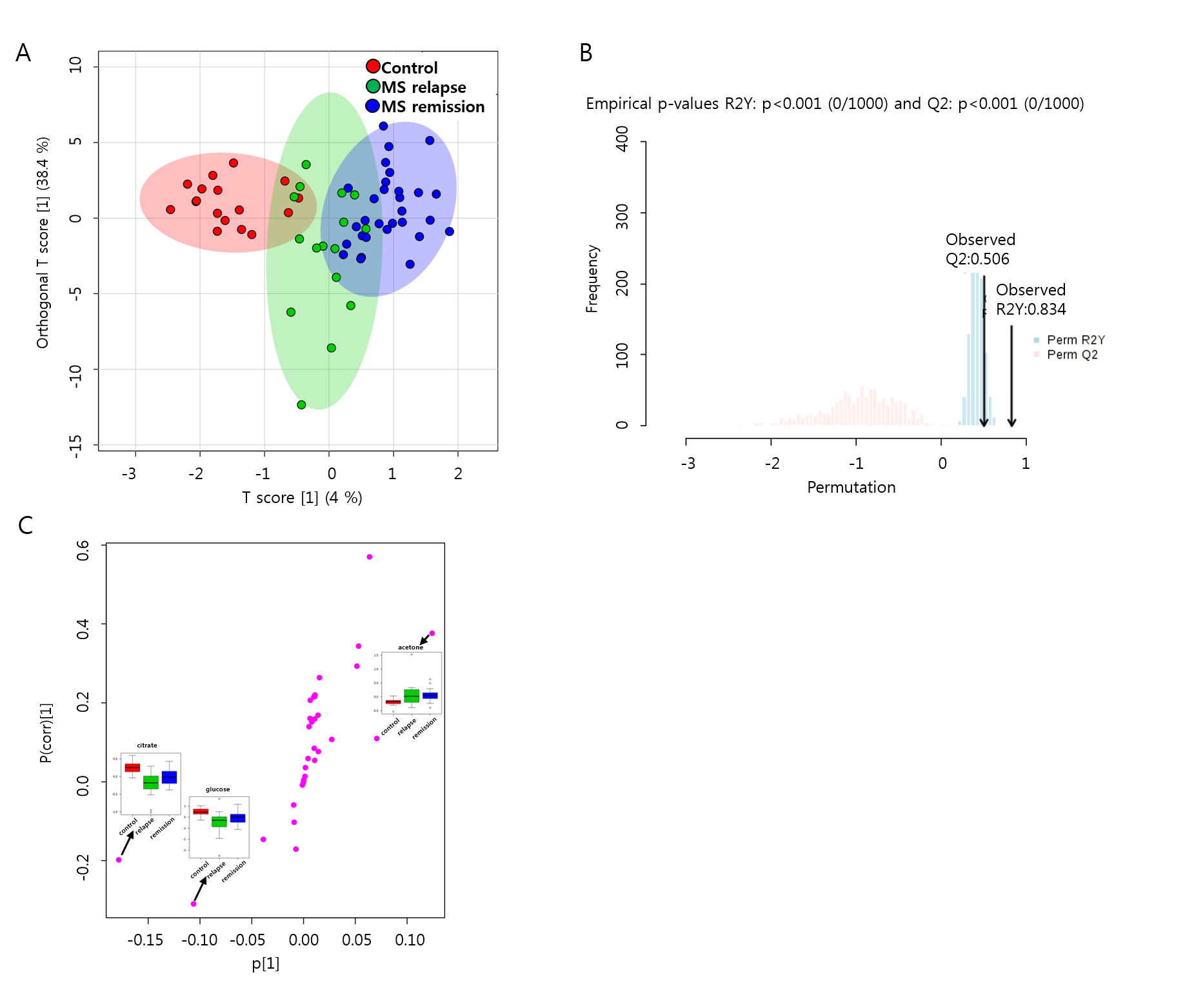

Supplement: S7 Fig — The OPLS-DA model was constructed and validated with CV-ANOVA and random permutation test. OPLS-DA scores plots of MS activity (A) are depicted. The red circles in (A) represent the control sample, the green circles represent the relapse patients, and the blue circles represent the remission patients. The 95% confidence ellipse of the group is depicted in each color. The Mahalanobis p-value between two groups (control–MS relapse, control–MS remission and MS relapse–MS remission) in OPLS-DA model for three groups were 1.072e-5, 5.926e-9 and 3.517e-2, respectively. The p-value of CV-ANOVA was 3.811e-5. (B) The permutation number was set to be 1,000. The observed R2 and Q2 values of the OPLS-DA model were higher than those obtained from the permuted tests, revealing predictability and goodness of fit. The permutation result of the OPLS-DA model for the three groups (control-MS relapse-MS remission) showed the empirical p-values of R2 (p < 0.001) and Q2 (p < 0.001). (C) S-plot of the OPLS-DA model. Influence of the model variable is shown. Glucose and citrate were relatively lowered in the relapse state compared to those in the remission state. Two metabolites as well as acetone seem to mainly contribute for group separation. (TIF) [file pone.0181758.s007.tif]
