# Supplementary material for: Metabolomic profiling of CSF in multiple sclerosis and neuromyelitis optica spectrum disorder by nuclear magnetic resonance
Source: PLoS One. 2017 Jul 26;12(7):e0181758. doi: 10.1371/journal.pone.0181758 (PMC5528902; doi:10.1371/journal.pone.0181758)
Supplement: S1 Table — Thirty-two metabolites were identified using the database stored in Chenomx NMR suite 7.7 and were quantified from the comparison of the internal standard (TSP). Data are presented as mean and standard deviation (SD). (DOCX) [file pone.0181758.s008.docx]

**S1 Table. The quantification of each metabolite (unit, mM)**

| **Metabolites** | **Healthy Control** | | **MS** | | **NMOSD** | |
| --- | --- | --- | --- | --- | --- | --- |
|  | **mean** | **SD^*^ (±)** | **mean** | **SD (±)** | **mean** | **SD (±)** |
| 2-hydroxybutyrate | 0.0420 | 0.0086 | 0.0544 | 0.0204 | 0.0718 | 0.0373 |
| 2-hydroxyisovalerate | 0.0044 | 0.0006 | 0.0044 | 0.0001 | 0.0053 | 0.0022 |
| 3-hydroxybutyrate | 0.0057 | 0.0010 | 0.0061 | 0.0013 | 0.0062 | 0.0011 |
| 3-hydroxyisobutyrate | 0.0072 | 0.0016 | 0.0074 | 0.0016 | 0.0076 | 0.0019 |
| 3-hydroxyisovalerate | 0.0038 | 0.0004 | 0.0035 | 0.0014 | 0.0046 | 0.0025 |
| acetate | 0.2993 | 0.0033 | 0.2626 | 0.0565 | 0.2563 | 0.0396 |
| acetoacetate | 0.0033 | 0.0006 | 0.0046 | 0.0048 | 0.0051 | 0.0023 |
| acetone | 0.1112 | 0.0364 | 0.1868 | 0.0864 | 0.2135 | 0.0627 |
| alanine | 0.0408 | 0.0051 | 0.0381 | 0.0110 | 0.0545 | 0.0409 |
| choline | 0.0021 | 0.0003 | 0.0025 | 0.0013 | 0.0028 | 0.0015 |
| citrate | 0.4352 | 0.0532 | 0.3451 | 0.0969 | 0.4698 | 0.1218 |
| creatine | 0.0541 | 0.0060 | 0.0513 | 0.0126 | 0.0531 | 0.0121 |
| creatinine | 0.0539 | 0.0027 | 0.0510 | 0.0092 | 0.0535 | 0.0108 |
| dimethyl sulfone | 0.0072 | 0.0012 | 0.0095 | 0.0144 | 0.0074 | 0.0019 |
| ethanol | 0.0127 | 0.0054 | 0.0199 | 0.0303 | 0.0202 | 0.0227 |
| fructose | 0.0565 | 0.0102 | 0.0533 | 0.0161 | 0.0517 | 0.0156 |
| formate | 0.0449 | 0.0061 | 0.0530 | 0.0101 | 0.0576 | 0.0197 |
| glucose | 4.5681 | 0.2540 | 4.0432 | 0.6704 | 4.1501 | 0.9873 |
| glutamine | 0.3291 | 0.0226 | 0.3203 | 0.0555 | 0.3434 | 0.0616 |
| hisitidine | 0.0081 | 0.0010 | 0.0071 | 0.0014 | 0.0077 | 0.0024 |
| isobutyrate | 0.0086 | 0.0019 | 0.0088 | 0.0019 | 0.0091 | 0.0023 |
| isoleucine | 0.0079 | 0.0013 | 0.0085 | 0.0030 | 0.0112 | 0.0056 |
| lactate | 1.8832 | 0.1715 | 1.8521 | 0.3670 | 2.4513 | 0.9397 |
| leucine | 0.0132 | 0.0020 | 0.0133 | 0.0041 | 0.0161 | 0.0065 |
| methanol | 0.0730 | 0.0185 | 0.0641 | 0.0213 | 0.0631 | 0.0413 |
| myo-inositol | 0.1651 | 0.0166 | 0.1548 | 0.0346 | 0.1569 | 0.0272 |
| phenylalanine | 0.0109 | 0.0012 | 0.0107 | 0.0023 | 0.0125 | 0.0035 |
| propylene glycol | 0.0073 | 0.0104 | 0.0049 | 0.0028 | 0.0065 | 0.0076 |
| pyroglutamate | 0.0332 | 0.0030 | 0.0454 | 0.0265 | 0.0443 | 0.0135 |
| pyruvate | 0.0085 | 0.0013 | 0.0093 | 0.0046 | 0.0103 | 0.0055 |
| tyrosine | 0.0107 | 0.0013 | 0.0109 | 0.0023 | 0.0123 | 0.0032 |
| valine | 0.0183 | 0.0033 | 0.0184 | 0.0060 | 0.0237 | 0.0120 |

^*^SD, standard deviation
